# Anti-Insulin Antibodies and Adverse Events with Biosimilar Insulin Lispro Compared with Humalog Insulin Lispro in People with Diabetes

**DOI:** 10.1089/dia.2017.0373

**Published:** 2018-02-01

**Authors:** Philip Home, Karl-Michael Derwahl, Monika Ziemen, Karin Wernicke-Panten, Suzanne Pierre, Yvonne Kirchhein, Satish K. Garg

**Affiliations:** ^1^ Institute for Cellular Medicine, Newcastle University, Newcastle upon Tyne, United Kingdom.; ^2^ Institut für Klinische Forschung und Entwicklung (IKFE), Berlin, Germany.; ^3^ Sanofi-Aventis Deutschland, Frankfurt, Germany.; ^4^ Sanofi, Paris, France.; ^5^ Barbara Davis Center for Diabetes, University of Colorado Denver, Aurora, Colorado.

**Keywords:** Anti-insulin antibodies, Biosimilar, Immunogenicity, Insulin lispro, SAR342434.

## Abstract

***Background:*** SAR342434 (SAR-Lis) is a biosimilar (follow-on) of insulin lispro (Humalog^®^; Ly-Lis). Two randomized, controlled, open-label, parallel-group, phase 3 studies were conducted to compare the efficacy and safety of SAR-Lis and Ly-Lis, both in combination with insulin glargine (Lantus^®^). SORELLA 1 was a 12-month study in 507 people with type 1 diabetes mellitus (T1DM); SORELLA 2 was a 6-month study in 505 people with type 2 diabetes mellitus (T2DM). In this study, the impact of anti-insulin antibodies (AIA) to SAR-Lis and Ly-Lis on safety and glycemic control is reported.

***Methods:*** AIA were measured regularly throughout both studies at a centralized laboratory blinded to treatment groups using a drug-specific AIA assay. The AIA status (positive or negative), AIA titers, and cross-reactivity to human insulin, insulin glargine, and insulin glargine metabolite M1 were analyzed. The potential effect of AIA on safety, particularly as related to hypersensitivity reactions, hypoglycemia, and treatment-emergent adverse events, as well as on glycemic control (HbA_1c_, insulin dose), was evaluated.

***Results:*** AIA positive status at baseline was similar for the two insulins, but higher in T1DM than in T2DM. In both studies, the percentage of people newly developing AIA in the two treatment groups, or having a ≥4-fold increase in AIA titers, did not differ. No relationship was observed between maximum individual AIA titers and change in HbA_1c_ or insulin dose, hypoglycemia, or hypersensitivity reactions or between efficacy/safety measures and subgroups by presence or absence of treatment-emergent AIA. Hypersensitivity events and events adjudicated as allergic reactions were few and did not differ between the two groups.

***Conclusion:*** Insulin lispro SAR342434 and the originator insulin lispro had a similar immunogenicity profile in people with T1DM or T2DM.

## Introduction

Insulin lispro is the active ingredient of Humalog^®^ (Eli Lilly, Indianapolis, IN), a rapid-acting insulin analog (100 U/mL) with faster onset and shorter duration of action than regular (unmodified) human insulin.^1^ Humalog (Ly-Lis) has been approved and marketed for use by people with type 1 (T1DM) or type 2 (T2DM) diabetes mellitus since 1996. SAR342434 (SAR-Lis; insulin lispro, Sanofi, Paris, France) has been developed as a biosimilar medicinal product to Humalog in the EU and as a follow-on product in the United States in accordance with the relevant EU and US guidelines.^2–6^ Similar pharmacokinetic (PK) and pharmacodynamic (PD) profiles were demonstrated for SAR-Lis to both EU-approved and US-approved Ly-Lis and between EU-approved and US-approved Ly-Lis in a PK/PD study in people with T1DM using the euglycemic clamp technique.^7^

No differences in efficacy and safety of SAR-Lis and Ly-Lis were found in two randomized (1:1), multinational, open-label, controlled, parallel-group, phase 3 studies in people with T1DM (SORELLA 1, 12 months) or T2DM (SORELLA 2, 6 months) on a background of insulin glargine (Lantus^®^; GLA-100, Sanofi) as basal insulin.^8,9^ In this study, we report on the immunogenicity profiles of participants from SORELLA 1 and SORELLA 2, and the potential impact of anti-insulin antibodies (AIA) on safety and efficacy in the SAR-Lis and Ly-Lis groups.

## Methods

The study design of SORELLA 1 (NCT02273180) and SORELLA 2 (NCT02294474), as well as participant selection criteria, disposition, baseline characteristics, and key efficacy and safety results, have been reported previously.^8,9^ A total of 1012 people (SORELLA 1, 507; SORELLA 2, 505) were randomized (1:1) to receive SAR-Lis (*N* = 506) or Ly-Lis (*N* = 506) along with GLA-100. Depending on the geographical area, people in the Ly-Lis group received US-approved Humalog (USA, Japan) or EU-approved Humalog (all other areas). Based on their similar PK/PD profiles, data from US- and EU-approved Ly-Lis were pooled within diabetes type in the comparator group of each study.

Randomization was stratified by HbA_1c_ at screening (<8.0, ≥8.0% [<64, ≥64 mmol/mol]), prior use of Humalog/Liprolog (Yes/No), and geographical region (non-Japan/Japan; SORELLA 1 only), and was performed centrally by an interactive voice response system/interactive web response system. SORELLA 1 involved 89 centers in Europe, Japan, and the United States, while SORELLA 2 involved 103 centers in Europe, South America, the United States, the Republic of Korea, and Turkey.

Men and women were eligible if ≥18 years old, with T1DM (SORELLA 1) or T2DM (SORELLA 2) diagnosed for at least 12 months. Criteria for participation included HbA_1c_ in the range of 7.0%−10.0% (53−86 mmol/mol; SORELLA 1) or 6.5%−10.0% (48−86 mmol/mol; SORELLA 2) and using once-daily GLA-100 as basal insulin and insulin lispro (Humalog/Liprolog) or insulin aspart (Novolog/NovoRapid; Novo Nordisk, Bagsværd, Denmark) as rapid-acting mealtime insulin at least thrice daily (before each meal) for at least 6 months.

Clinical visits were scheduled at screening, randomization (day 1), and weeks 4, 8, 12, 20, and 26 (SORELLA 1 and SORELLA 2), and in SORELLA 1 additionally at weeks 40 and 52. SAR-Lis or Ly-Lis was self-administered by subcutaneous injection with an insulin pen within 5–10 min before the start of a main meal (breakfast, lunch, and dinner) and any additional meals and snacks. Details of the protocols have been described previously.^8,9^

Ethical approval according to local regulations was obtained for all study sites. Conduct of the studies adhered to standards of data collection for clinical trials, according to the Declaration of Helsinki. Written informed consent was obtained from all participants before starting the studies.

### Immunogenicity

Blood samples for determination of AIA were drawn at least 8 h after last administration of SAR-Lis or Ly-Lis at day 1 and week 4, 12, and 26 in both studies, and at week 40 and 52 in SORELLA 1. Anti-SAR-Lis antibodies were analyzed at a central laboratory blinded to the treatment group, employing a quasi-quantitative radioimmunoprecipitation assay. The assay was validated in accordance with recent recommendations applying screening, confirmatory, titer, and cross-reactivity cutpoints.^10^ Cross-reactivity cutpoints were validated for human insulin, insulin glargine, and insulin glargine metabolite M1. The sensitivity of the assay was 22.1 μg/L; thus the assay was capable of detecting anti-insulin lispro antibodies with high sensitivity.

The samples were analyzed for AIA status (positive or negative), titers, and, due to the high amino acid sequence homology, AIA cross-reactive to insulin glargine, glargine M1 metabolite, and human insulin. The analysis of AIA data focused on the change in AIA response observed following the administration of the investigational medicinal product (IMP) using the following definitions^11^: treatment-emergent AIAs defined as treatment-induced or treatment-boosted AIAs; treatment-induced AIAs defined as AIAs found de novo (seroconversion) following the IMP administration or AIAs detected during IMP administration in participants with missing baseline sample; and treatment-boosted AIAs defined as preexisting AIAs that were boosted to an at least fourfold increase in AIA titer compared with baseline at any time following the IMP administration. A fourfold increase in titer corresponds to two titer steps, while a single titer step would be within the expected imprecision of the titration method. The analyses were done for AIA titers (1/dil), representing a quasi-quantitative expression of the level of AIA in a sample (as recommended in current literature^10^). The results are presented for the 12-month period of SORELLA 1 and the 6-month period of SORELLA 2.

The incidence of AIA was defined as all participants with “treatment-boosted” or “treatment-induced” AIA (i.e., participants with treatment-emergent AIA), while the prevalence of AIA was defined as all participants with at least one positive AIA sample during the study (baseline or treatment emergent). Peak titers were defined as the maximal individual titers observed during the on-treatment period. A transient response was defined as a response detected only once at one sampling time, except the last, during the on-treatment period, or responses detected more often, but where the first and last AIA-positive samples were <16 weeks apart, and the last sampling time point was AIA negative.

A persistent response was a response detected at least twice, where the first and last positive samples were ≥16 weeks apart, or responses detected at the last two sampling time points irrespective of the time period in between them. An indeterminate response was a response where only the last sample was positive. AIA outliers were defined as AIA titer levels higher than 1.5 times the interquartile range above the third quartile of distribution of peak AIA titers, corresponding in both studies to AIA titers ≥64 (1/dil).

An Allergic Reaction Assessment Committee (ARAC) was convened, which consisted of three people who were specialists in allergy and clinical immunology and one was a specialist in diabetology with past interest in insulin immunogenicity. Hypersensitivity reactions identified by Medical Dictionary for Regulatory Activities (MedDRA) search and potential allergic reactions reported by the investigator on the dedicated allergic reaction form were reviewed by the ARAC members who were blinded to the study treatment. For events confirmed as allergic, the ARAC proposed a diagnosis and assessed the possible relationship to IMP and the severity of the event.

The ARAC also reviewed reports of participants with AIA titers elevated over the baseline level at the study endpoints and ongoing hypersensitivity treatment-emergent adverse events (TEAEs, defined as events that occurred, worsened, or became serious from first IMP dose up to 1 day after last IMP dose) and/or ongoing serious adverse events (SAEs), and/or an increase of HbA_1c_ >1.0% (>11 mmol/mol) above baseline, and/or an unexplained increase in insulin dose (defined as total daily insulin dose >1.5 U/kg or insulin dose increase >20% above baseline [SORELLA 1] and >2.0 U/kg or insulin dose increase >70% above baseline [SORELLA 2]). The ARAC assessed if any of these conditions was suspected to be AIA mediated and provided a recommendation on the need for follow-up of these participants.

### Statistical analysis

Statistical methods for both studies have been reported previously.^8,9^ Immunogenicity analyses were descriptive and based on the AIA population, defined as all participants from the safety population (all participants randomized and exposed to at least one dose of SAR-Lis or Ly-Lis) with at least one AIA sample available for analysis during the on-treatment period (from first IMP dose up to 1 day after last IMP dose). Boxplots were provided to assess AIA titers over time.

The potential impact of immune response on safety and efficacy endpoints was assessed using subgroup analyses of HbA_1c_ change from baseline to study end, hypoglycemia, injection site reactions, hypersensitivity reactions, TEAEs, and SAEs by treatment-emergent AIA (Yes/No). Descriptive statistics were provided for each subgroup. Least square (LS) means for the HbA_1c_ analysis by treatment-emergent AIA were obtained from a mixed-effect model for repeated measures with treatment group, randomization strata of screening HbA_1c_ and prior use of Humalog/Liprolog, visit, treatment-by-visit interaction, AIA subgroup, AIA subgroup-by-treatment interaction, AIA subgroup-by-visit interaction, and AIA subgroup-by-visit-by-treatment interaction as fixed categorical effects, and baseline HbA_1c_ value and baseline HbA_1c_ value-by-visit interaction as continuous fixed covariates.

The significance level of the AIA subgroup-by-treatment interaction was provided for descriptive purposes to assess potential heterogeneity of the treatment effect across AIA subgroups. For hypoglycemia, the significance level of the AIA subgroup-by-treatment interaction was also provided for descriptive purposes based on a logistic regression model with fixed-effect terms for treatment, randomization strata of screening HbA_1c_ and prior use of Humalog/Liprolog, AIA subgroup, and AIA subgroup-by-treatment interaction. Scatterplots were also performed to assess the relationship between the individual maximal AIA titers during the on-treatment period and HbA_1c_ change from baseline to study end, total insulin dose, hypoglycemia, and hypersensitivity reactions. All analyses were conducted using SAS Enterprise Guide version 5.1 (SAS Institute, Cary, NC).

## Results

In SORELLA 1, 507 people were randomized, of whom 506 received the investigation insulin (SAR-Lis, 252; Ly-Lis, 254). A total of 505 people were randomized and treated in SORELLA 2 (SAR-Lis, 253; Ly-Lis, 252). Demographic data were similar between the SAR-Lis and Ly-Lis groups in both studies and details of these two studies are provided elsewhere.^8,9^

Overall efficacy and safety results in the studies did not differ between the two insulin treatment groups.^8,9^ Non-inferiority of SAR-Lis vs Ly-Lis on HbA_1c_ change from baseline at the prespecified 0.30% (0.33 mmol/mol) non-inferiority margin and inverse non-inferiority were demonstrated at week 26 in both SORELLA 1 (LS mean difference of SAR-Lis vs. Ly-Lis: 0.06% [95% CI: −0.08 to 0.20]) and SORELLA 2 (−0.07% [−0.22 to 0.07]). The 6-month extension period for SORELLA 1 demonstrated that similar glycemic control was maintained with SAR-Lis and Ly-Lis at similar insulin dosages over 1 year.^8^ Changes from baseline to study end in the daily mealtime and basal insulin dose were small and similar for the two treatment groups within both studies.^8,9^ Hypoglycemia and TEAEs did not differ between groups in either study.^8,9^

### AIA status

The AIA population consisted of 500 participants (SAR-Lis, 248; Ly-Lis, 252) in SORELLA 1 and 493 participants (SAR-Lis, 245; Ly-Lis, 248) in SORELLA 2. Similar percentages of participants in the two treatment groups were positive for AIA at baseline, with higher percentages in SORELLA 1 (SAR-Lis, 47.6%; Ly-Lis, 49.2%) than SORELLA 2 (SAR-Lis, 24.5%; Ly-Lis, 25.4%) (Table 1).

**Table 1.** Summary of Anti-Insulin Antibody Response During the On-Treatment Period in SORELLA 1 and SORELLA 2 – Anti-Insulin Antibody Populations

	*SORELLA 1 (12 months)*		*SORELLA 2 (6 months)*	
	*SAR-Lis (*N* = 248)*	*Ly-Lis (*N* = 252)*	*SAR-Lis (*N* = 245)*	*Ly-Lis (*N* = 248)*
Participants with AIA positive at baseline, *n* (%)	118/248 (47.6)	124/252 (49.2)	60/245 (24.5)	63/248 (25.4)
Median titer (1/dil)	4.00	4.00	4.00	4.00
Q1:Q3	2.00:16.00	2:00:8.00	2.00:8.00	2.00:16.00
Participants with ≥4-fold increase in titer (treatment boosted), *n* (%)	19/118 (16.1)	26/124 (21.0)	12/60 (20.0)	8/63(12.7)
Median peak titer^a^ (1/dil)	16.00	16.00	12.00	16.00
Q1:Q3	8.00:32.00	16.00:32.00	8.00:32.00	8.00:32.00
Transient AIA response, *n* (%)	0/19	0/26	0/12	0/8
Persistent AIA response, *n* (%)	19/19 (100)	26/26 (100)	12/12 (100)	8/8 (100)
Indeterminate AIA response, *n* (%)	0/19	0/26	0/12	0/8
Participants with AIA negative or missing at baseline, *n* (%)	130/248 (52.4)	128/252 (50.8)	185/245 (75.5)	185/248 (74.6)
Participants newly positive postbaseline (treatment induced), *n* (%)	37/130 (28.5)	35/128 (27.3)	34/185 (18.4)	28/185 (15.1)
Median peak titer^a^ (1/dil)	2.00	2.00	2.00	2.00
Q1:Q3	1.00:4.00	1.00:4.00	1.00:8.00	1.00:4.00
Transient AIA response, *n* (%)	10/37 (27.0)	10/35 (28.6)	8/34 (23.5)	8/28 (28.6)
Persistent AIA response, *n* (%)	21/37 (56.8)	21/35 (60.0)	15/34 (44.1)	10/28 (35.7)
Indeterminate AIA response, *n* (%)	6/37 (16.2)	4/35 (11.4)	11/34 (32.4)	10/28 (35.7)
Participants with at least one positive AIA sample (prevalence)^b^, *n* (%)	155/248 (62.5)	159/252 (63.1)	94/245 (38.4)	91/248 (36.7)
Participants with treatment-emergent AIA (incidence)^c^, *n* (%)	56/248 (22.6)	61/252 (24.2)	46/245 (18.8)	36/248 (14.5)
Participants without treatment-emergent AIA, *n* (%)	192/248 (77.4)	191/252 (75.8)	199/245 (81.2)	211/248 (85.1)
Inconclusive participants, *n* (%)	0/248	0/252	0/245	1/248 (0.4)

AIA, anti-insulin antibody; dil, dilution; Ly-Lis, insulin lispro; SAR-Lis, SAR243424.

^a^ Maximal titer measured during the on-treatment period.

^b^ Prevalence: participants AIA positive at baseline or with treatment-induced AIAs.

^c^ Incidence: participants with treatment-boosted or treatment-induced AIAs (i.e., participants with treatment-emergent AIAs).

For definition of transient, persistent, and indeterminate responses, see Methods section.

**FIG. 1.**
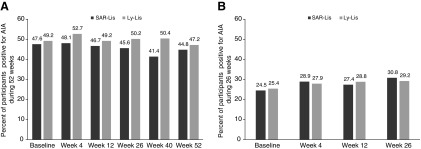
Percentage of participants positive for AIA during 52-week on-treatment period of SORELLA 1 **(A)** and 26-week on-treatment period of SORELLA 2 **(B)**/AIA populations. AIA, anti-insulin antibody; Ly-Lis, insulin lispro; SAR-Lis, SAR342434.

Similar percentages of participants in the SAR-Lis and Ly-Lis groups were positive for AIA at least at one time point (prevalence) between baseline (inclusive) and week 52 in SORELLA 1 (62.5% and 63.1%) and between baseline (inclusive) and week 26 in SORELLA 2 (38.4% and 36.7%). The percentage of participants in the SAR-Lis and Ly-Lis groups positive for AIA remained relatively stable during the on-treatment period in SORELLA 1, being 44.8% and 47.2%, respectively, at week 52 (Fig. 1A). In SORELLA 2, the percentages of participants positive for AIA were also similar between the SAR-Lis and Ly-Lis groups at week 26, with 30.8% and 29.2%, respectively (Fig. 1B).

**FIG. 2.**
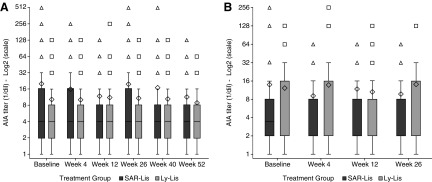
Boxplots of AIA titer (1/dil) over time during the 52-week on-treatment period in participants with T1DM (SORELLA 1) **(A)** and the 26-week on-treatment period in participants with T2DM (SORELLA 2) **(B)**/AIA populations. AIA, anti-insulin antibody; Ly-Lis, insulin lispro; SAR-Lis, SAR342434; T1DM, type 1 diabetes mellitus; T2DM, type 2 diabetes mellitus. At each visit, AIA titers are described for participants with a positive-sample AIA status at the visit. The boxplot provides the 25% (Q1), 50% (median), and 75% (Q3) quartiles (lower, middle, and upper horizontal bars of the box, respectively). The diamond represents the mean, and triangles or squares represent values beyond the upper/lower whiskers (defined as 1.5 times the interquartile range). Each symbol for high/low values could represent more than one participant.

Median AIA titers were similar at baseline (4.00) between the two treatment groups in the two studies and remained relatively unchanged over time with an interquartile range of 2.00–16.00 in the SAR-Lis group and 2.00–8.00 in the Ly-Lis group in SORELLA 1 (Fig. 2A) and 2.00–8.00 in the SAR-Lis group and 2.00–16.00 in the Ly-Lis group in SORELLA 2 (Fig. 2B). Maximum AIA titers in T1DM participants throughout the study (baseline included) were 512 (1/dil) in the SAR-Lis group and 256 (1/dil) in the Ly-Lis group, and were 256 (1/dil) in both treatment groups in T2DM participants. For SAR-Lis in T2DM participants, the maximum of 256 was at baseline; it was 64 postbaseline.

When only people taking insulin lispro (Humalog, US or EU or Liprolog) before the study were considered, the proportion “positive” at baseline was similar to that in the total population (above) being 51.4% in SORELLA 1 and 22.7% in SORELLA 2 (Supplementary Table S1; Supplementary Data are available online at www.libertpub.com/dia). Prevalence in the SORELLA 1 study was 65.8% for SAR-Lis and 62.0% for Ly-Lis, and in SORELLA 2, 35.4% for SAR-Lis and 33.3% for Ly-Lis. AIA titers remained relatively low in both treatment groups over the study period in both studies.

### Treatment-emergent AIA

The overall percentages of participants with a treatment-emergent AIA response (incidence) were similar between the SAR-Lis and Ly-Lis treatment groups during the 12-month on-treatment period in SORELLA 1 (22.6% and 24.2%) and the 6-month on-treatment period in SORELLA 2 (18.8% and 14.5%) (Table 1). Treatment-boosted AIA were found in 16.1% of participants in the SAR-Lis group and 21.0% in the Ly-Lis group in SORELLA 1 and in 20.0% and 12.7% of participants in SORELLA 2. The median of the individual peak titers for participants with treatment-boosted AIAs were 16 (1/dil) in the two treatment groups in SORELLA 1 and 12 (1/dil) in the SAR-Lis group and 16 (1/dil) in the Ly-Lis group in SORELLA 2. In both studies, the AIA response was persistent in all participants with treatment-boosted AIAs (Table 1).

A similar percentage of participants in the SAR-Lis and Ly-Lis groups had treatment-induced AIA in both SORELLA 1 (28.5% and 27.3%) and SORELLA 2 (18.4% and 15.1%) (Table 1). For these participants, the median of the individual peak titers was 2 (1/dil) in both treatment groups in the two studies, while maximum titers were 32 (1/dil) in both treatment groups in SORELLA 1 and 64 (1/dil) in the SAR-Lis group and 128 (1/dil) in the Ly-Lis group in SORELLA 2. Differences between the SAR-Lis and Ly-Lis groups were minimal in both studies, and the percentage of participants with a transient AIA response was similar in both treatment groups (SORELLA 1: SAR-Lis 27.0%, Ly-Lis 28.6%; SORELLA 2: SAR-Lis 23.5%, Ly-Lis 28.6%) (Table 1).

When only participants taking insulin lispro (Humalog, US or EU, or Liprolog) before the study were considered, the proportions with treatment-emergent AIAs were similar to the total study populations (above), being 21.9% and 22.8% for SAR-Lis and Ly-Lis in SORELLA 1, and 16.2% and 15.1% in SORELLA 2 (Supplementary Table S1).

### Cross-reactivity

The proportion of participants with antibodies cross-reacting with human insulin at each visit was similar between the two groups and ranged between 87.2% and 94.7% during the 12-month on-treatment period in SORELLA 1 and between 80.3% and 96.8% during the 6-month on-treatment period in SORELLA 2. The proportion of participants with antibodies cross-reacting with GLA-100 or insulin glargine metabolite M1 at each visit ranged from 84.4% to 92.7% and 71.0% to 79.7%, respectively, in SORELLA 1 and from 81.0% to 89.9% and 65.2% to 85.5%, respectively, in SORELLA 2.

### Influence of AIA on efficacy and safety outcomes

**FIG. 3.**
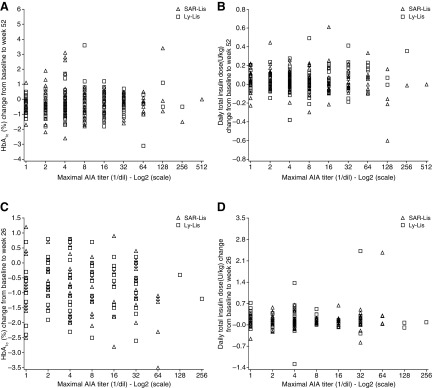
Scatterplots of the change from baseline in HbA1c **(A, C)** and total insulin dose **(B, D)** to week 52 in SORELLA 1 **(A, B)** and to week 26 in SORELLA 2 **(C, D)** by maximum individual AIA titers/AIA populations. Ly-Lis, insulin lispro; SAR-Lis, SAR342434.

The mean change in HbA_1c_ from baseline to study end in SORELLA 1 and SORELLA 2 was similar for the SAR-Lis and Ly-Lis groups in both participants with and without treatment-emergent AIA (Table 2). The treatment-by-treatment-emergent-AIA interaction showed no heterogeneity of treatment effect across the two subgroups of AIA status in SORELLA 1 (*P* = 0.42) or SORELLA 2 (*P* = 0.69). There was no relationship observed between the change in HbA1c and the individual maximal AIA titers in either study (Fig. 3A, C).

**Table 2.** Summary of HbA_1c_ and Insulin Dose for Participants with and Without Treatment-Emergent Anti-Insulin Antibodies in SORELLA 1 and SORELLA 2/Anti-Insulin Antibody Populations

	*Treatment-emergent AIA*							
	*SORELLA 1 (52 weeks)*				*SORELLA 2 (26 weeks)*			
	*Yes*^a^		*No*		*Yes*^a^		*No*	
	*SAR-Lis (*N* = 56)*	*Ly-Lis (*N* = 61)*	*SAR-Lis (*N* = 192)*	*Ly-Lis (*N* = 191)*	*SAR-Lis (*N* = 46)*	*Ly-Lis (*N* = 36)*	*SAR-Lis (*N* = 199)*	*Ly-Lis (*N* = 211)*
HbA_1c_ (%)								
Baseline	8.18 (0.74)	7.97 (0.64)	8.05 (0.79)	8.01 (0.64)	7.92 (0.87)	7.92 (0.93)	8.02 (0.86)	8.04 (0.91)
Week 52/26	7.92 (1.05)	7.66 (0.91)	7.77 (0.96)	7.72 (0.98)	7.08 (0.83)	7.25 (1.02)	7.05 (0.86)	7.15 (0.86)
LS mean (SE) change from baseline to week 52/26	–0.14 (0.12)	–0.33 (0.11)	–0.25 (0.07)	–0.29 (0.07)	–0.88 (0.12)	–0.74 (0.13)	–0.93 (0.06)	–0.85 (0.06)
LS mean difference^b^ (SE) vs. Ly-Lis [95% CI]	0.19 (0.17) [−0.14 to 0.52]		0.04 (0.09) [−0.14 to 0.22]		−0.15 (0.18) [−0.49 to 0.20]		−0.07 (0.08) [−0.23 to 0.09]	
Mean daily insulin dose (U/kg)								
Basal insulin								
Baseline	0.34 (0.16)	0.31 (0.14)	0.34 (0.21)	0.34 (0.14)	0.46 (0.22)	0.52 (0.22)	0.48 (0.27)	0.44 (0.23)
Week 52/26	0.45 (0.82)	0.36 (0.15)	0.36 (0.23)	0.34 (0.16)	0.54 (0.24)	0.57 (0.25)	0.56 (0.32)	0.52 (0.27)
Change from baseline to week 52/26	0.14 (0.78)	0.04 (0.07)	0.02 (0.07)	0.00 (0.06)	0.07 (0.09)	0.07 (0.09)	0.08 (0.14)	0.07 (0.13)
Mealtime insulin								
Baseline	0.39 (0.18)	0.35 (0.13)	0.36 (0.18)	0.36 (0.18)	0.44 (0.27)	0.47 (0.28)	0.46 (0.30)	0.43 (0.32)
Week 52/26	0.38 (0.15)	0.35 (0.17)	0.38 (0.18)	0.36 (0.17)	0.49 (0.24)	0.56 (0.49)	0.53 (0.35)	0.50 (0.41)
Change from baseline to Week 52/26	0.01 (0.12)	0.01 (0.10)	0.02 (0.12)	0.01 (0.11)	0.04 (0.16)	0.10 (0.28)	0.10 (0.22)	0.08 (0.24)
Total insulin								
Baseline	0.73 (0.29)	0.66 (0.22)	0.70 (0.32)	0.70 (0.25)	0.90 (0.37)	0.99 (0.40)	0.94 (0.49)	0.86 (0.45)
Week 52/26	0.72 (0.22)	0.72 (0.29)	0.73 (0.30)	0.70 (0.27)	1.02 (0.35)	1.14 (0.64)	1.09 (0.60)	1.02 (0.57)
Change from baseline to week 52/26	0.03 (0.17)	0.05 (0.12)	0.04 (0.13)	0.01 (0.13)	0.12 (0.21)	0.18 (0.30)	0.18 (0.31)	0.15 (0.30)

Data are mean (SD) unless otherwise stated.

LS, least square; SAR-Lis, SAR342434; SE, standard error.

^a^ Participants with preexisting AIAs that were boosted to a significant higher titer (at least fourfold increase) compared to baseline, or participants without preexisting AIA (or missing baseline) and with at least one positive AIA sample.

^b^ Mixed-effect model for repeated measures with treatment group (SAR-Lis, Ly-Lis), randomization strata of screening HbA_1c_ (<8.0, ≥8.0%) and prior use of Humalog/Liprolog (Yes, No), visit (week 12, 26, as well as week 40 and 52 for SORELLA 1), treatment-by-visit interaction, AIA subgroup, AIA subgroup-by-treatment interaction, AIA subgroup-by-visit interaction, and AIA subgroup-by-visit-by-treatment interaction as fixed categorical effects, and baseline HbA_1c_ value and baseline HbA_1c_ value-by-visit interaction as continuous fixed covariates.

Daily doses for basal, mealtime, and total insulin were generally comparable across treatment groups and were independent of the presence of treatment-emergent AIA (Table 2). The mean dose changes from baseline to week 52 (SORELLA 1) and week 26 (SORELLA 2) did not suggest use of higher insulin doses in participants with treatment-emergent AIA compared with those without treatment-emergent AIA, and in the context of the HbA_1c_, findings do not suggest specific unmet need. No relationship was observed between the individual maximal AIA titers and the change in total daily insulin dose from baseline to end of treatment in the two studies (Fig. 3B, D).

**FIG. 4.**
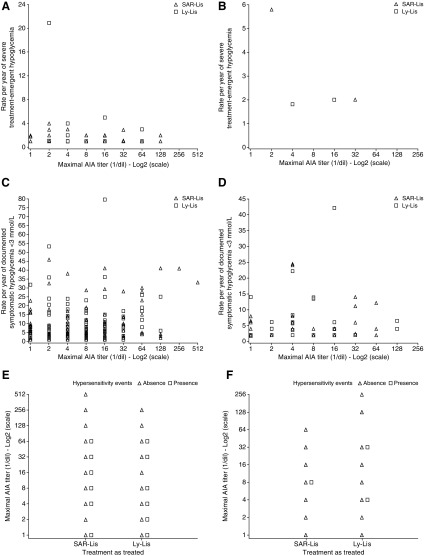
Scatterplots of the rate of severe hypoglycemia **(A, B)** and documented symptomatic hypoglycemia (plasma glucose <3 mmol/L [54 mg/dL]) **(C, D),** and the presence/absence of hypersensitivity reactions **(E, F)** by maximal individual AIA titers in SORELLA 1 **(A, C, E)** and SORELLA 2 **(B, D, F)** during the on-treatment period of each study/AIA populations. Note: One participant in SORELLA 1 with 122 episodes of severe hypoglycemia was excluded in A (see Results section). Ly-Lis, insulin lispro; SAR-Lis, SAR342434.

In both studies, the number of participants with AIA titers ≥64 (1/dil) for treatment-emergent AIAs was small and similar in both treatment groups (SORELLA 1: SAR-Lis, four participants; Ly-Lis, five participants; SORELLA 2: SAR-Lis, two participants; Ly-Lis, one participant). The review of HbA_1c_, insulin doses, and safety parameters (hypersensitivity event and hypoglycemia) in these participants did not suggest negative effects of high AIA titers on these measures in either treatment group. There was no relationship observed in either study between the individual maximum AIA titers and the rate of hypoglycemia events per year (shown for severe hypoglycemia and documented symptomatic hypoglycemia [<3.0 mmol/L; 54 mg/dL]) or hypersensitivity events (Fig. 4).

In participants with treatment-emergent AIA, severe hypoglycemia was reported by 10 people (17.9%) in the SAR-Lis group (1−4 events per person and one person with 122 events discussed below) and six people (9.8%) in the Ly-Lis group (1−5 events per person) in SORELLA 1 (Table 3). In most episodes of severe hypoglycemia, the symptoms reported by participants were weakness/palpitation/increased sweating/nervousness/confusion/headache/dizziness/tremor.

**Table 3.** Event Table for Severe Hypoglycemia, Hypersensitivity Reactions, Injection Site Reactions, Treatment-Emergent Adverse Events, and Serious Adverse Events with Treatment-Emergent Anti-Insulin Antibodies in SORELLA 1 and SORELLA 2 – Anti-Insulin Antibody Populations

	*SORELLA 1 (52 weeks)*				*SORELLA 2 (26 weeks)*			
	*Treatment-emergent AIA*				*Treatment-emergent AIA*			
	*Yes*^a^		*No*		*Yes*^a^		*No*	
	*SAR-Lis (*N* = 56)*	*Ly-Lis (*N* = 61)*	*SAR-Lis (*N* = 192)*	*Ly-Lis (*N* = 191)*	*SAR-Lis (*N* = 46)*	*Ly-Lis (*N* = 36)*	*SAR-Lis (*N* = 199)*	*Ly-Lis (*N* = 211)*
Hypoglycemia, *n* (%)								
Severe hypoglycemia	10 (17.9)	6 (9.8)	23 (12.0)	28 (14.7)	0	1 (2.8)	6 (3.0)	2 (0.9)
Hypersensitivity reactions and injection site reactions, *n* (%)								
Any hypersensitivity reactions	1 (1.8)	2 (3.3)	14 (7.3)	14 (7.3)	1 (2.2)	1 (2.8)	8 (4.0)	8 (3.8)
Any injection site reactions	0	2 (3.3)	3 (1.6)	1 (0.5)	1 (2.2)	1 (2.8)	0	3 (1.4)
TEAEs and SAEs, *n* (%)								
Any TEAE	30 (53.6)	32 (52.5)	106 (55.2)	109 (57.1)	21 (45.7)	14 (38.9)	92 (46.2)	92 (43.6)
Any SAE	4 (7.1)	6 (9.8)	16 (8.3)	13 (6.8)	2 (4.3)	4 (11.1)	10 (5.0)	21 (10.0)

^a^ Participants with preexisting AIAs that were boosted to a significant higher titer (at least fourfold increase) compared to baseline, or participants without preexisting AIA (or missing baseline) and with at least one positive AIA sample.

TEAE, treatment-emergent adverse event; SAE, serious adverse event; SAR-Lis, SAR34243.

One person in the SAR-Lis group reported 122 hypoglycemia events (119 reported during the first 6 months of the study) requiring assistance in the form of oral carbohydrates and thus classed as severe hypoglycemia. None of the hypoglycemia was reported as requiring other forms of carbohydrate administration, glucagon, or other resuscitative actions. In none of the episodes in this person were severe neuroglycopenic symptoms reported. The site did not believe all events truly required assistance. Notably, HbA_1c_ had improved markedly in this person during the study (baseline: 9.4%; week 26: 7.4%) and AIAs were detectable at week 12 only (titer: 1 [1/dil]).

In SORELLA 2, no one in the SAR-Lis group and one participant in the Ly-Lis group reported severe hypoglycemia among participants with treatment-emergent AIA (Table 3). In those participants without treatment-emergent AIAs, severe hypoglycemia was reported by 23 people (12.0%) in the SAR-Lis group (1−4 events per person) and 28 people (14.7%) in the Ly-Lis group (1−5 events per person) in SORELLA 1, and by 6 people (3.0%) in the SAR-Lis group (1 event per person) and 2 people (0.9%) in the Ly-Lis group (1 event per person) in SORELLA 2. The treatment-by-treatment-emergent-AIA interaction showed no heterogeneity of treatment effect for any hypoglycemia category.

In both treatment groups in both studies, hypersensitivity events were reported by few participants with treatment-emergent AIAs. In both studies, the incidence of hypersensitivity reactions was greater in both treatment groups in participants without treatment-emergent AIAs compared with the group of participants with treatment-emergent AIAs (Table 3). Across both studies, TEAEs were adjudicated by ARAC as allergic reactions in four participants (0.8%) in the SAR-Lis group (seasonal allergy, contact dermatitis, allergic rhinitis, and allergy to arthropod bite) and four participants (0.8%) in the Ly-Lis group (urticaria, allergic rhinitis, pruritus, and mouth swelling). None of the allergic reactions in either study was considered related to the investigational insulin and none was suspected to be AIA mediated. Injection site reactions occurred in very few participants: 0–3 participants/group in both studies regardless of AIA status.

In SORELLA 1, the percentage of participants with any TEAE was similar in the SAR-Lis and Ly-Lis groups in those with (53.6% and 52.5%, respectively) or without (55.2% and 57.1%, respectively) treatment-emergent AIA (Table 3). In SORELLA 2, the percentage of participants with any TEAE was slightly higher in the SAR-Lis group (45.7%) than in the Ly-Lis group (38.9%) in people with treatment-emergent AIA and similar between the groups in people without treatment-emergent AIA (SAR-Lis, 46.2%; Ly-Lis, 43.6%), with no single TEAE accounting for the difference. The most frequently reported TEAEs at the primary system organ class (SOC) level were infections and infestations in both SORELLA 1 (SAR-Lis, 32.5%; Ly-Lis, 31.9%) and SORELLA 2 (SAR-Lis, 20.2%; Ly-Lis, 16.3%). TEAEs in the other SOCs were reported in less than 10%–11% of participants regardless of treatment group.

The percentage of participants with serious TEAEs was also similar in the two treatment groups in SORELLA 1 regardless of AIA status, with slightly higher percentages in people without treatment-emergent AIA (Table 3). In SORELLA 2, the percentage of participants with serious TEAEs was higher in the Ly-Lis group than the SAR-Lis group with (11.1% vs. 4.3%) or without (10.0% vs. 5.0%) treatment-emergent AIA, with no single SAE accounting for the difference.

There were 13 cases in SORELLA 1 (SAR-Lis, 5; Ly-Lis, 8) and four cases in SORELLA 2 (SAR-Lis, 1; Ly-Lis, 3) with AIA titers elevated above the baseline value at study endpoint and concomitant ongoing hypersensitivity events or potential indicators of deterioration of glycemic control. The ARAC did not suspect these cases to be AIA mediated and did not deem it necessary to follow up with the people in any of the cases.

## Discussion

Regulatory guidelines for approval of biosimilar (follow-on) pharmaceuticals, mainly proteins and including insulin, generally require clinical studies of immunogenicity lasting 1 year notably in the United States (as mandated by the U.S. Food and Drug Administration).^2,12^ This can be traced to problems with some biosimilar products in the past because pharmaceutical proteins are potentially antigenic in humans,^13^ and because immunogenicity is one of the ways to detect impurities left over from manufacturing. Indeed for insulin, in particular up to the 1970s, immunological problems were not uncommon, something that can be traced to the continued presence of insulin and proinsulin derivatives rather than the small changes in amino acid sequence of the then animal insulins.^14^

However with the introduction of chromatographic manufacturing procedures, immunological insulin resistance and injection site changes decreased significantly. Low titers of insulin antibodies are usual, however, even in those exposed only to human insulin. This seems to be related to the development of low levels of insulin derivatives in the late stages of manufacturing, and storage of insulin thereafter.^14^ Furthermore, many people with diabetes, especially T1DM, have detectable levels of insulin antibodies as part of the pathological process of the condition, even before they receive insulin products.^15^

Thus, as in this study, sensitive assays will uncover low serum levels of insulin antibodies, even in those taking, or only exposed to, modern insulin analogues. The proportion of people found to be “positive” will vary with the sensitivity of the assay, and here in people with T1DM, this approaches 50%, although lower in people with T2DM, with their different human leukocyte antigen profile. This is consistent with previous studies for lispro insulin, which also noted that lispro insulin performed similarly in terms of antibody status to human insulin.^16^

When exposed to SAR-Lis compared with Ly-Lis, the changes in antibody profile, whether viewed as those with treatment-emergent positivity, a clinically significant rise in titers, or those with higher titers, did not differ, implying that the immunogenicity of SAR-Lis did not differ from Ly-Lis. Adjudicated changes in glucose control (as HbA_1c_) or insulin dose (i.e., to detect immunological insulin resistance in occasional individuals) failed to detect any single case on either insulin. Further the distribution of changes in HbA_1c_ and insulin dose implies no relationship to insulin antibody titers even at the highest titers seen, implying that the sensitivity of the assay is such that the antibody levels detected even on dilution to 1:64 or higher are too low to influence insulin absorption, insulin availability in the plasma or extracellular space, or insulin-receptor interactions.

There are statistical limitations to our approach, indeed generally in the insulin antibody area. As noted above, the situation differs from exposure to a new biopharmaceutical (such as an anti-tumor necrosis factor agent) because at baseline, our populations have high percentages of “positive” individuals, with the median around the limit of detection. The phenomenon of regression to the mean will then indicate that any subpopulation (such as those deemed “negative” or “positive” in line with consensus recommendations^10^) will tend to the original distribution on retesting, such that there will be a high conversion in either direction even without any intervention.

In insulin studies, there is always an intervention even in the control group, but, in a high proportion of those randomized, Lilly lispro was swapped to Ly-Lis. Nevertheless, in these participants, an apparent insulin antibody response was seen in a large proportion of those previously “negative”, and enhanced levels in others. It seems likely that this is simple regression toward the original distribution. This also explains why the apparent prevalence of positivity increased during the study, more and more individuals having the chance of at least one positive result.

As in those randomized from Lilly lispro to SAR-Lis, the findings are very similar to those randomized from Lilly lispro to Ly-Lis; the implication is that there was no detectable immunological effect of the biosimilar lispro beyond that of prior insulins. Since the findings for those coming into the study from Lilly lispro are close to those for the whole study, the implication is that the background antigenicity for those coming off other analogues (mainly insulin aspart) was also the same both before and during the study and independent of exposure to SAR-Lis or Ly-Lis.

It should be noted that as well as statistical effects, concurrent medical conditions, such as infections or autoimmune disease, might be expected to boost insulin antibody titers in a few individuals as part of more general immunological activation. However, while the numbers are small, there is no imbalance between the SAR-Lis and Ly-Lis treatment groups in “treatment-boosted” titers.

Other immunological phenomena related to insulin therapy include skin reactions rarely and hypersensitivity reactions extremely rarely. No signal for any difference for these was found in our studies, but the expected incidence would be too low to detect even quite a large change, given the population exposures we used. Hypoglycemia has been related to very high antibody titers notably in neonates.^14^ In SORELLA 1, numbers of participants with severe hypoglycemia in the overall population were 34 in both treatment groups, and too small to be meaningful in SORELLA 2. No other immunologically or non-immunologically related adverse events differed between the treatment groups.

We conclude that the biosimilar (follow-on) insulin lispro SAR342434 and the originator insulin lispro had a similar immunogenicity profile in people with T1DM and T2DM. They have no relevant association with surrogate clinical outcomes in either population.

## Supplementary Material

Supplemental data
