# Supplementary material for: Anti-Insulin Antibodies and Adverse Events with Biosimilar Insulin Lispro Compared with Humalog Insulin Lispro in People with Diabetes
Source: Diabetes Technol Ther. 2018 Feb 1;20(2):160–70. doi: 10.1089/dia.2017.0373 (PMC5771536; doi:10.1089/dia.2017.0373)
Supplement: Supplemental data [file Supp_Table1.pdf]

Supplementary Data

Supplementary Table S1. Summary of Anti-Insulin Antibody Response in Participants Using Insulin Lispro Before SORELLA 1 (12 Months) and SORELLA 2 (6 months) – AIA Populations

	SORELLA 1		*SORELLA 2*	
Participants	*SAR-Lis* (N = *155)*	*Ly-Lis* (N = *158)*	*SAR-Lis* (N = *130)*	*Ly-Lis* (N = *126)*
AIA positive at baseline, *n* (%)	80/155 (51.6)	81/158 (51.3)	31/130 (23.8)	27/126 (21.4)
Median titer (1/dil)	8.00	4.00	4.00	4.00
Q1:Q3	2.00:16.00	2.00:12.00	2.00:8.00	2.00:8.00
Treatment-boosted AIA, *n* (%)	12/80 (15.0)	19/81 (23.5)	6/31 (19.4)	4/27 (14.8)
Median peak titer[a] (1/dil)	16.00	16.00	8.00	24.00
Q1:Q3	12.00:96.00	8.00:32.00	8.00:32.00	12.00:32.00
AIA negative or missing at baseline, *n* (%)	75/155 (48.4)	77/158 (48.7)	99/130 (76.2)	99/126 (78.6)
Treatment-induced AIA, *n* (%)	22/75 (29.3)	17/77 (22.1)	15/99 (15.2)	15/99 (15.2)
Median peak titer[a] (1/dil)	1.00	2.00	4.00	2.00
Q1:Q3	1.00:4.00	1.00:4.00	1.00:32.00	1.00:4.00
Participants with ≥1 positive AIA sample (prevalence)[b], *n* (%)	102/155 (65.8)	98/158 (62.0)	46/130 (35.4)	42/126 (33.3)
Patients with treatment-emergent AIA (incidence)[c], *n* (%)	34/155 (21.9)	36/158 (22.8)	21/130 (16.2)	19/126 (15.1)

[a]Maximal titer measured during the on-treatment period.
[b]Prevalence: participants AIA positive at baseline or with treatment-induced AIA.
[c]Incidence: participants with treatment-boosted or treatment-induced AIAs (i.e., participants with treatment-emergent AIA).
AIA, anti-insulin antibodies; Ly-Lis, insulin lispro; SAR-Lis, SAR342434.
